# Genetic aspects of idiopathic scoliosis – literature review

**DOI:** 10.1186/1748-7161-7-S1-O71

**Published:** 2012-01-27

**Authors:** P Janusz, T Kotwicki, K Małgorzata, A Szulc

**Affiliations:** 1 Spine Disorders Unit Department of Pediatric Orthopedics and Traumatology University of Medical Sciences, Poznan, Poland; 2 Department of Cell Biology University of Medical Sciences, Poznan, Poland

## Purpose of the study

The purpose of this study was to review relevant literature describing polymorphisms of genes associated to idiopathic scoliosis. Etiology of idiopathic scoliosis (IS) remains unrevealed, however genetic background is strongly advocated. Loci on chromosomes and genes polymorphisms associated to occurrence and progression of IS were the subject of research.

## Materials and methods

Medline, Embase and Cochrane Library electronic databases were searched from 1990 till 2010. The search was limited to English language. The following key words were used: idiopathic scoliosis, gene, genetics, chromosome. Based on the abstract, the relevance of each article was assessed, then full-text articles were obtained [1-5].

## Results

558 abstracts were identified, 51 full texts were obtained and 18 were analyzed. The genes polymorphisms with the evidence of association to occurrence or progression of IS were identified. Both the papers confirming or denying genetic background of IS were found. The genes presenting polymorphisms susceptible to be related to IS were as follows: estrogen receptors (ER), melatonin receptors (MTNR), matrilin (MATNI), chromodomain helicase DNA-binding protein (CHD7), interleukin-6 (IL-6), metalloproteinases (MMP-3), γ1-syntrophin (SNTG1), aggrecan, tryptophan hydroxylase 1 (TPH1), arylalkylamine N-acetyltransferase (AANAT), growth hormone receptor (GHR), collagen and elastic fibers genes. Up to now seven of the above mentioned could be excluded while others are subject of further investigation.

## Conclusion

There is still insufficient data about the genetic origin and development of IS. The amount and quality of the existing publications increases, suggesting possible discovery of genetic background and understanding of molecular course of IS.
